# Blood type A associated with critical COVID-19 and death in a Swedish cohort—a critical comment

**DOI:** 10.1186/s13054-020-03264-z

**Published:** 2020-09-04

**Authors:** Joern Bullerdiek

**Affiliations:** 1 Institute of Medical Genetics, Medical Center, University of Rostock, D-18057 Rostock, Germany; 2 Human Genetics, University of Bremen, D-28359 Bremen, Germany

I read with great interest the letter by Hultström et al. entitled “Blood type A associates with critical COVID-19 and death in a Swedish cohort” [1]. The authors report an association of blood group A with the risk of requiring critical care as well as with an increased risk of death within 30 days. The study is based on the blood group distribution from a Swedish critical care cohort (*n* = 64) compared to the blood type distribution in the population as a whole. It is stated that this latter study confirms earlier papers on blood type and COVID-19 severity such as a genome-wide association study by Ellinghaus et al. [2] and a clinical/serological study by Zietz and Tatonetti [3] both identifying blood group A as a risk factor for disease severity and death in COVID-19. Nevertheless, the letter gives rise to concerns.

First, the paper suffers from its control group, i.e., the general population. The findings do not allow for the conclusion that AB0 blood types influence the severity of symptoms since no comparison has been made between patients suffering from severe COVID-19 disease versus those only mildly affected (e.g., non-hospitalized) as a control group. Associations with blood type thus can also be expected if they affect one’s chance getting infected at all.

Secondly, the study by Zietz and Tatonetti, neither in its first nor in the second version of the preprint [3], does allow for the conclusion that on average blood group A coincides with a more severe course of COVID-19. According to the first version, the study “did not identify any significant relationships between blood group and intubation or death due to COVID-19.” As to the second version with more than two thousand individuals tested positive (COV+), the only significant AB0 blood group association was between blood group A and intubation but even with an opposite direction (odds ratio 0.762, 95% confidence interval [0.620–0.937], *p* = 0.0099).

Thirdly, another important study in the field [4] on 1289 COV+ individuals with a known blood type has not been cited. This study did not reveal an association between blood type and the risk of intubation or death.

In summary, the study by Hultström et al. should be interpreted with some caution.

## Data Availability

All data mentioned are in the public domain. No additional own data or unpublished 3rd party data were used.

## Abbreviation

COV+: Individuals tested positive for SARS-CoV-2
